# Cardiorespiratory dose comparison among six radiotherapy regimens for patients with left-sided breast cancer

**DOI:** 10.1038/s41598-023-40577-9

**Published:** 2023-08-16

**Authors:** Yongkai Lu, Yanfang Ma, Di Yang, Yi Li, Wei Yuan, Fengwen Tang, Lei Xu, Luping Zhou, Hao Lin, Binglin Li, Ruijuan Chen, Chenchen He, Dongli Zhao

**Affiliations:** 1 Department of Radiation Oncology, The First Affiliated Hospital of Xi’an Jiaotong University, No.277, Yanta West Road, Xi’an, 710061 Shaanxi China; 2 Department of Radiation Oncology, Shaanxi Provincial Tumor Hospital, Affiliated Hospital of Xi’an Jiaotong University Health Science Center, Xi’an, China; 3 Department of Thoracic Surgery, Xi’an Central Hospital, Xi’an, China

**Keywords:** Cancer, Cardiology, Oncology

## Abstract

There is uncertainty regarding the benefits and drawbacks of various radiation protocols for the treatment of left-sided breast cancer. To address this issue, we conducted a Bayesian network analysis. First, we searched several electronic databases for eligible literature. Next, we pooled the data from twelve studies concerning three-dimensional conformal radiation therapy (3D-CRT), intensity modulated radiation therapy (IMRT), and volumetric modulated arc therapy (VMAT), combined with either deep inspiratory breath-holding (DIBH) or free-breathing (FB) modalities. The integrated cardiac and pulmonary dosimetric indexes for all included treatments were compared using Bayesian networks. A direct meta-analysis indicated that for the two methods of 3D-CRT and IMRT, DIBH technology was more effective than FB in reducing the radiation dose to the heart and lungs. Additionally, according to the network results, DIBH was superior to FB in all six treatment options, regardless of whether the plan was 3D-CRT, IMRT, or VMAT. Besides, the combined data indicated that the FB-3D-CRT regimen had the weakest dosimetric advantage of all the treatments. Excluding FB-3D-CRT, each of the other five treatments had its own specific benefits. This is the first Bayesian study of several radiotherapy regimens for breast cancer patients on the left side, and the findings can be used to select appropriate radiotherapy programs for breast cancer patients.

## Introduction

Concerning the treatment of early breast cancer, radiation therapy has become a crucial component of the overall therapeutic strategy. Besides, it is widely agreed that breast cancer radiation therapy dramatically improves overall survival^1,2^. In recent decades, the number of long-term survivors has increased due to various medical advances^3^. However, as the rate of survival grows, the likelihood of suffering a variety of late radiation-related adverse effects increases. Darby et al*.* demonstrated that the incidence of severe coronary events caused by radiation rose linearly with mean heart dose (MHD) by 7.4% per gray, with no threshold dose^4^. Clarke et al*.* compared a group of irradiated patients to a group of non-irradiated patients and discovered a considerably higher death rate, particularly for heart disease and lung cancer, with rate ratios of 1.27 and 1.78, respectively^5^.

Thus, for patients who receive radiotherapy for breast cancer, substantial efforts have been made to develop techniques that reduce the dose of radiation to the heart and lungs, such as deep inspiration breath-holding (DIBH). This simple technique reduces cardiac exposure through lung expansion, which physically displaces the heart away from the treatment field. There are several approaches for performing DIBH, such as active breath control, external infrared box markers, and optical surface monitoring^6^. Studies have demonstrated that for left-sided breast cancer patients, DIBH reduces the cardiac dose compared with free-breathing (FB^7–11^. It is worth mentioning that the DIBH technique has high repeatability and stability across the whole treatment process^12^.

Most recent reports on the application of DIBH vary in the choice of the treatment plan. Three-dimensional conformal radiation therapy (3D-CRT), intensity modulated radiation therapy (IMRT), and volumetric modulated arc therapy (VMAT) have all been utilized, but there are no comprehensive comparisons of these three methods. Additionally, an extensive systematic review into the differences in left-sided breast cancer radiotherapy between the DIBH and FB groups has not yet been performed. Network meta-analysis, also known as multiple-treatments comparison, enables the synthesis of data from both direct (within-trial) and indirect comparisons (inter-trial treatment comparisons through a common comparator treatment) of diverse regimens^13^. The Bayesian approach allows us to estimate the rank probability that each of the treatments is the best, second best, etc.^14^. Therefore, in this study, we sought to provide useful information regarding the comparisons between these six radiotherapy regimens through integrated and indirect methods. We expect the findings of this study to be helpful for physicians and patients when deciding on treatment options.

## Materials and methods

### Search methodology

We searched PubMed, EMBASE, the Cochrane library, and the Web of Science using the search phrases "breast cancer", "radiotherapy", and "deep inspiration breath-hold", with a deadline of September 15, 2022). There was no constraint on the language of the studies that were published. Additionally, three investigators independently conducted literature searches and screening as well as a manual evaluation of the references of the chosen studies. Disagreements were settled through discussion with a fourth investigator.

### Inclusion criteria

The PICOS guiding principles (Participants, Intervention, Comparison, Outcomes, and Study design) were applied in all the included investigations. The inclusion criteria were: (1) Participants [P]: Patients were identified pathologically with left-sided breast carcinoma devoid of distant metastases. Following breast-conserving surgery, the prescribed dose of whole-breast irradiation was 50 Gy in 25 fractions; (2) Intervention [I]: Patients in the experimental group were administered DIBH. The treatment techniques used were VMAT, IMRT, and 3D-CRT; (3) Comparison [C]: In the control group, the intervention was the free-breathing (FB) regimen combined with the three therapies of VMAT, IMRT, and 3D-CRT. It should be noted that the geometric distribution of the field is described differently in the literature, but the basic principle is the classical tangential field arrangement, especially for the 3D-CRT and IMRT planning approaches, and for the VMAT approach the start and end angles of the field must cover as little lung tissue as possible while meeting the target area coverage; (4) Outcomes [O]: The outcomes were dosimetric indicators of the heart, left anterior descending artery, and ipsilateral lung, including the mean dose (D_mean_) and the proportion of the ipsilateral lung volume receiving at least 20 Gy (V20).These metrics will be the vehicle for comparing different radiotherapy techniques; (5) Study design [S]: randomized controlled trials (RCTs) and observational studies, including cohort and case–control studies.

### Exclusion criteria

Articles that met any of the following criteria were rejected: (1) Review articles, case studies, correspondence, and abstracts; (2) Reports with poor research quality or a strong potential for bias; (3) Articles lacking data that could be aggregated.

### Data extraction

Two researchers (Mr. Li and Mr. Yang) independently retrieved the following information from the included studies: First author, year of publication, country, study design, patient age, DIBH type, clinical tumor stage, sample size, detailed treatment plan, and outcomes of the various subgroups. Disputes concerning data extraction were arbitrated by a third investigator (Ms. Yuan).

### Quality evaluation

To evaluate the bias potential in nonrandomized research, the Newcastle–Ottawa Scale (NOS) was employed, comprising three dimensions: selection, comparability, and outcomes^15^. With total scores ranging from 0 to 9, 4 points were awarded for selection, 2 points for comparability, and 3 points for outcomes. Studies scoring at least 6 points were deemed good quality^16^.

### Statistical analysis

To synthesize papers comparing the same pair of treatments, pair-wise meta-analyses were conducted using RevMan software version 5.4 (Cochrane Collaboration, Oxford, UK). To evaluate measurement data, the standardized mean difference (SMD) and 95% confidence interval (95% CI) were used as the effect indicators. The assessment of heterogeneity across trials was conducted using the Cochrane Q test and the *I*^2^ statistic, which provided a measure of the percentage of total variability attributable to heterogeneity rather than random error. In instances where the P-value of the Q test exceeded 0.10 and the *I*^2^ value was less than 50%, a fixed-effects model was employed to analyze data that exhibited non-significant heterogeneity^17^. Values of P < 0.01 were considered statistically significant. Next, we constructed a random-effects network using the Markov Chain Monte Carlo (MCMC) method in GeMTC 0.14.3 within a Bayesian framework. When the MCMC reached a stage of stable convergence, estimations and inferences were performed. The GeMTC parameters were set as follows: the initial value was set to 2.5; the number of simulation iterations was 50,000; 20,000 adjustment iterations were first performed to eliminate the influence of the initial value; the step size (sparse interval) was set to 10 when the number of chains was 4. The potential scale reduced factor (PSRF) reflected the convergence of the model, and when the PSRF value approached 1 (indicating satisfactory convergence), the homogeneity model was regarded as consistent enough for further research. Finally, the ranking likelihood for each intervention was calculated and the node-splitting approach was utilized to assess local inconsistency^18^.

## Results

### Study selection

**Figure 1:**
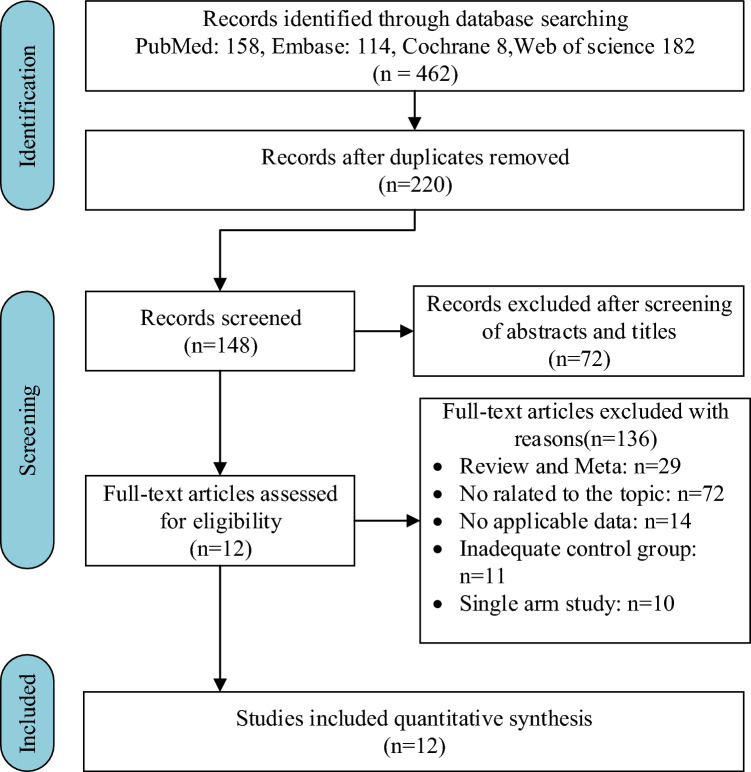
Flow chart of the search process for the meta-analysis.

After removing duplicates, preliminary searches in PubMed, Embase, the Cochrane Library, and Web of Science yielded 220 original studies. According to the initial screening of titles and abstracts, 29 papers were deemed eligible. Following an examination of the entire texts of these reports, 17 articles were removed for the following reasons: (1) publication of duplicate data; (2) lack of valid data; (3) publication as conference abstracts. Following the inclusion and exclusion criteria, twelve studies^8,19–29^ were ultimately included in this network meta-analysis. Figure 1 depicts the flowchart of the selection process.

### Study characteristics

The twelve studies in the meta-analysis^8,19–29^ involved 714 left-sided breast cancer patients. All included articles were retrospective studies determined to be of high quality, according to the Newcastle–Ottawa Scale^15^. Table 1 provides a summary of the baseline data for the twelve included studies. When multiple groups of data were included in the same study, each data group had to be counted separately.

**Table 1:** Characteristics of the studies included in the meta-analysis.

First author (year of publication)	Total patients (DIBH/FB)	Clinical stage	Median age (years)	Prescription dose (Gy)/fractions (F)	Plan types	Study type	NOS score
Angela 2017^21^	64 (32/32)	NA	NA	50 Gy/25 F	3D-CRT	Retrospective	6
Bruzzaniti 2013^27^	16 (8/8)	NA	51	50 Gy/25 F	3D-CRT	Retrospective	7
Chi. F. 2015^26^	62 (31/31)	I or II	39.5	50 Gy/25 F	IMRT	Retrospective	8
Hepp 2015^25^	40 (20/20 )	pTis–pT1 pN0	NA	50 Gy/25 f	3D-CRT	Retrospective	7
Jensen 2017^23^	44 (22/22)	pT1-2N0M0	58	50 Gy/25 f	3D-CRT	Retrospective	7
Lastrucci 2017^22^	46 (23/23)	NA	NA	50 Gy/25 f	3D-CRT	Retrospective	7
Corradini 2017^29^ (3D-CRT group)	20 (10/10)	NA	NA	50 Gy/25 f	3D-CRT	Retrospective	7
Corradini 2017^29^ (VMAT group)	20 (10/10)	NA	NA	50 Gy/25 f	VMAT	Retrospective	7
Pham 2016^24^ (IMRT group)	30 (15/15)	NA	NA	50 Gy/25 f	IMRT	Retrospective	6
Pham 2016^24^ (VMAT group)	30 (15/15)	NA	NA	50 Gy/25 f	VMAT	Retrospective	6
Sakyanun 2020^19^	50 (25/25)	NA	NA	50 Gy/25 f	IMRT	Retrospective	6
Vikström 2011^28^	34 (17/17)	NA	60	50 Gy/25 f	3D-CRT	Retrospective	6
Yamauchi 2020^8^	170 (85/85)	NA	49.3	50 Gy/25 f	IMRT	Retrospective	7
Zhao-Feng 2018^20^ (3D-CRT Group)	44 (22/22)	NA	48	50 Gy/25 f	3D-CRT	Retrospective	7
Zhao-Feng 2018^20^ (IMRT group)	44 (22/22)	NA	48	50 Gy/25 f	IMRT	Retrospective	7

*DIBH* deep inspiration breath hold, *FB* free breathing, *NOS* Newcastle–Ottawa Scale, *VMAT* volumetric modulated arc therapy, *IMRT* intensity-modulated radiation therapy, *3D-CRT* 3-dimensional conformal radiotherapy, *NA* not available.

### Direct meta-analysis

**Figure 2:**
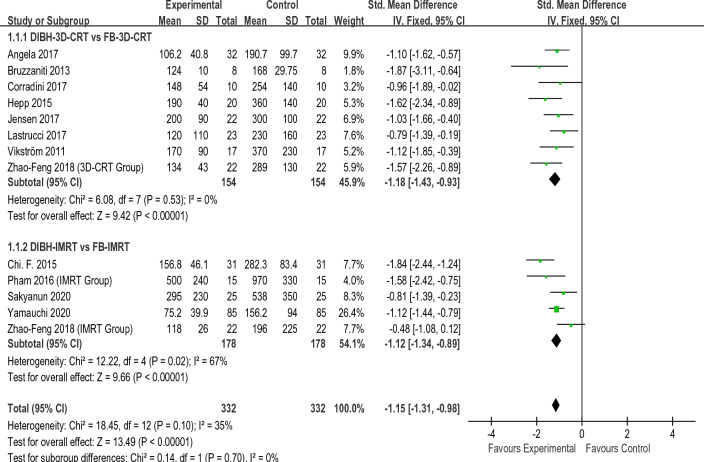
Direct meta-analyses of heart mean dose.

**Figure 3:**
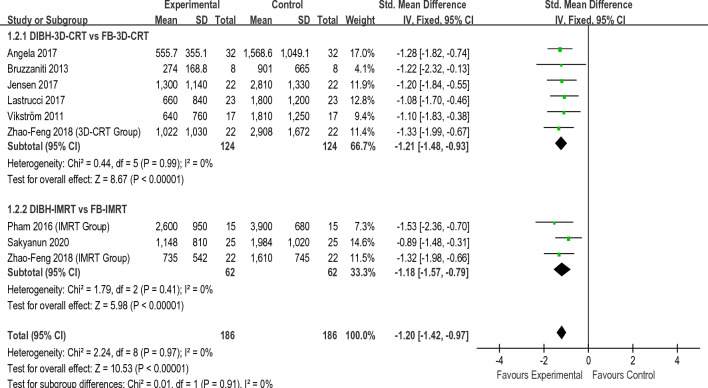
Direct meta-analyses of LAD mean dose.

**Figure 4:**
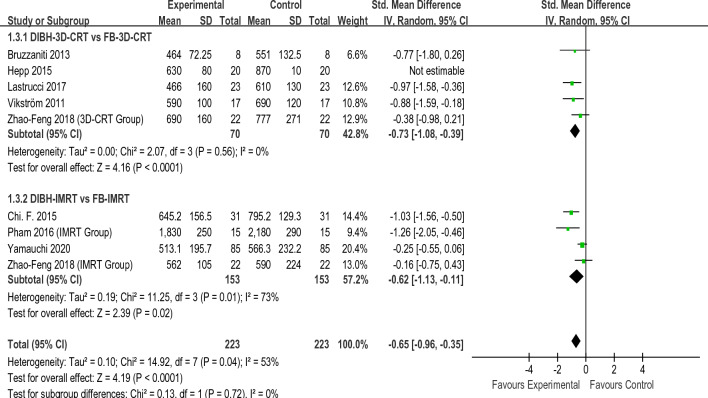
Direct meta-analyses of lung mean dose.

**Figure 5:**
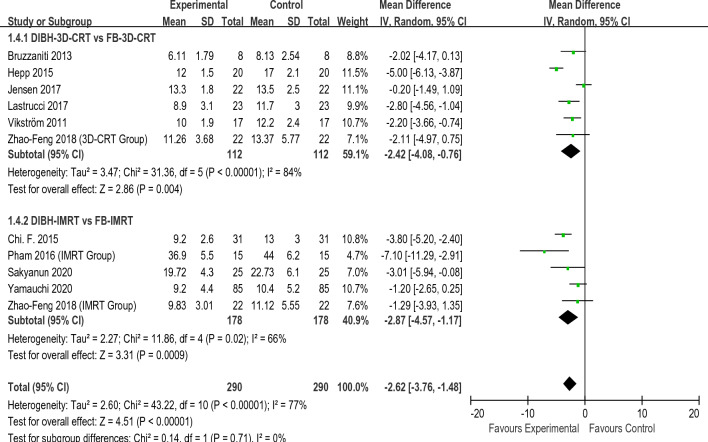
Direct meta-analyses of lung V20.

Figures 2, 3, 4, 5 present the direct meta-analysis results of heart mean dose, left anterior descending (LAD)mean dose, ipsilateral lung mean dose, and ipsilateral lung V20, respectively. Heart mean dose data were extracted from all twelve articles^8,19–29^, comprising 664 patients. Since between-study heterogeneity was negligible (*I*^2^ < 50%, P ≥ 0.10), we applied a fixed-effects model. The pooled results indicated that there was a substantial difference between the DIBH-3D-CRT and FB-3D-CRT groups, as well as between the DIBH-IMRT and FB-IMRT groups. Eight studies^19–24,27,28^ involving 372 patients were eligible for LAD mean dose analysis. No significant heterogeneity was identified (*I*^2^ < 50%, P ≥ 0.10), so a fixed-effects model was employed to calculate the pooled data. Results revealed that the average dose of LAD in the DIBH-3D-CRT group was significantly lower than in the FB-3D-CRT group. An identical situation also appeared in the comparison between the DIBH-IMRT group and the FB-IMRT group. Ipsilateral lung mean dose data were extracted from eight studies^8,20,22,24–28^ comprising 446 patients. The heterogeneity test revealed statistically significant differences among the studies (*I*^2^ ≥ 50%, P ≤ 0.10), therefore, a random-effects model was applied. The mean dose to the ipsilateral lung in the DIBH-3D-CRT group was lower than that of the FB-3D-CRT group, and the dose in the DIBH-IMRT group was also lower than the FB-IMRT group. Ten studies^8,19,20,22–28^ were appropriate for analyzing ipsilateral lung V20. We employed a random-effects model because a significant difference was observed in the heterogeneity test (*I*^2^ ≥ 50%, P ≤ 0.10). The results showed that the V20 value of the DIBH-3D-CRT group was lower than that of the FB-3D-CRT group, and the performance of the DIBH-IMRT group was also better than that of the FB-IMRT group.

In summary, by combining the results with the clinical information from the included studies, we realized that in the two methods of 3D-CRT and IMRT, the DIBH approach was more effective than FB in reducing heart mean dose, LAD mean dose, ipsilateral lung mean dose, and ipsilateral lung V20. It should be noted that there were only two studies that explored VMAT, so data merging and direct meta-analysis were not possible. Table 2 shows the summary results of the direct meta-analysis.

**Table 2:** Summary results of direct meta-analysis

Indicators	Comparative groups	Models	95% CI	P-value
Heart mean dose	DIBH-3D-CRT VS FB-3D-CRT	Fixed	− 1.18 [− 1.43, 0.93]	< 0.01
	DIBH-IMRT VS FB-IMRT		− 1.12 [− 1.34, − 0.89]	< 0.01
LAD mean dose	DIBH-3D-CRT VS FB-3D-CRT	Fixed	− 1.21 [− 1.48, − 0.93]	< 0.01
	DIBH-IMRT VS FB-IMRT		− 1.18 [− 1.57, − 0.79]	< 0.01
Lung mean dose	DIBH-3D-CRT VS FB-3D-CRT	Random	− 0.73 [− 1.08, − 0.39]	< 0.01
	DIBH-IMRT VS FB-IMRT		− 0.62 [− 1.13, − 0.11]	< 0.01
lung V20	DIBH-3D-CRT VS FB-3D-CRT	Random	− 2.42 [− 4.08, − 0.76]	< 0.01
	DIBH-IMRT VS FB-IMRT		− 2.87 [− 4.57, − 1.17]	< 0.01

### Networks for multiple treatment comparisons

**Figure 6:**
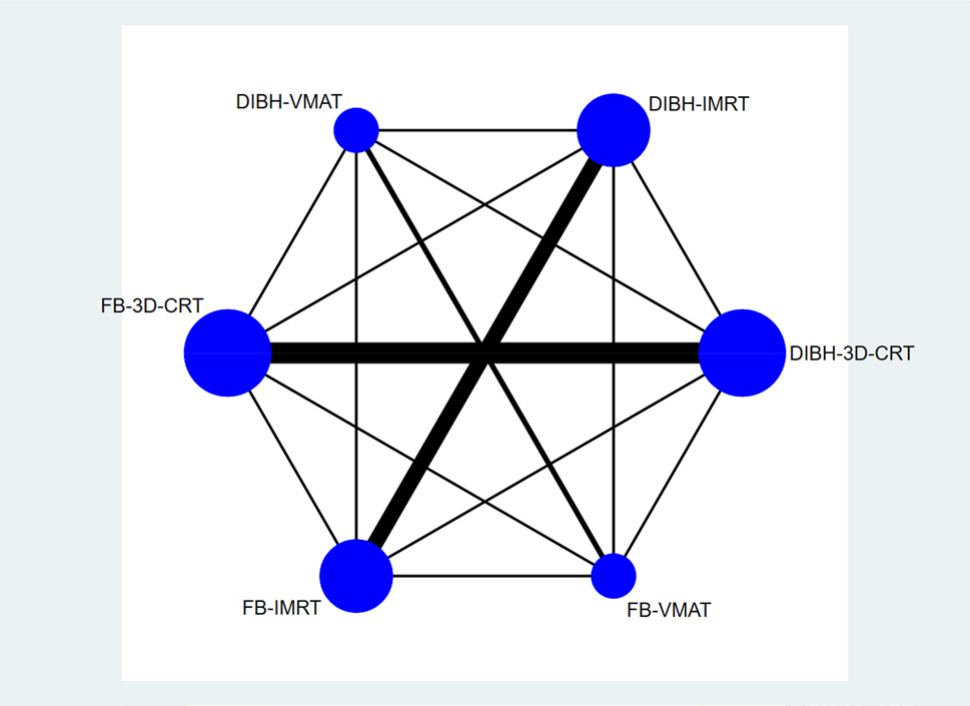
Network established for multiple treatment comparisons (the graph's points are proportional to the sample number of interventions, and the lines' thickness is proportional to their association).

A network map of the six interventions was generated using Stata 15.0, as Fig. 6 shows. The size of the points in the graph represents the weight of the sample number of interventions, and the thickness of the lines in the figure is proportional to the correlation between the two interventions. The figure indicates that DIBH-3D-CRT and FB-3D-CRT were the two most effective strategies in this study. DIBH-IMRT and FB-IMRT were the next most effective, DIBH-VMAT and FB-VMAT were the least. It is important to note that Fig. 6 denotes a measure based on mean cardiac dose, which signifies that there are direct pairwise comparisons between all protocols. However, the network graph is not closed for the other three metrics involved in this study. As a result, a network meta-analysis was performed to combine direct comparisons with indirect comparisons.

### Network meta-analyses

Table 3 summarized the results of the multiple-treatments meta-analyses regarding heart mean dose, LAD mean dose, ipsilateral lung mean dose and ipsilateral lung V20 according to network. Statistically significant results are shown in bold in Table 3. According to the network results, the choice of deep inspiratory breath-holding for respiratory management with a fixed radiotherapy technique (3D-CRT, IMRT, or VMAT) had better results. Coherence between direct and indirect comparisons based on networks was confirmed. In terms of heart mean dose, the network analysis results do not support the comparison of the advantages and disadvantages of the three regimens of FB-3D-CRT, FB-IMRT and FB-VMAT, but the results show that the average cardiac dose of DIBH-3D-CRT is lower than that of FB-IMRT and FB-VMAT. In addition, the results also showed that the mean cardiac dose of DIBH-IMRT was lower than that of FB-3D-CRT, FB-IMRT and FB-VMAT, but the mean heart dose of DIBH-3D-CRT compared with DIBH-IMRT did not show an advantage. Finally, Bayesian analysis showed that DIBH-VAMT was only superior to the FB-VAMT regimen in terms of mean cardiac dose, with insignificant differences with the other four regimens; For the LAD mean dose, the results showed that the FB-3D-CRT group had higher values than all the other five groups, while the DIBH-VMAT had lower values than the other five groups. We might conclude that for the average dose of LAD, the DIBH-VMAT scheme is the best choice and the FB-3D-CRT is the worst choice. In addition, the results also showed that the FB-VMAT regimen was the best choice in the free breathing group, because the average dose of LAD in this regimen was both smaller than that of the FB-IMRT squid FB-3D-CRT; Regarding the mean dose in the ipsilateral lung, the results showed no statistically significant difference between FB-3D-CRT and FB-IMRT, but FB-3D-CRT had a disadvantage compared with the other four groups; Regarding the ipsilateral lung V20 indicator, the Bayesian analysis results show that DIBH-3D-CRT scheme is not only inferior to DIBH-VMAT, but also worse than FB-IMRT, which has never been reported in previous studies. In addition, the analysis results of V20 indicators showed FB-3D-CRT may still be the least optional of the six solutions.

**Table 3:** Multiple treatment comparison for dosimetry indicators based on network (bolded bold indicates that the pair of comparisons is statistically significant).

Heart mean dose					
DIBH-3D-CRT					
** − 114.45 (− 179.15, − 59.03)**	FB-3D-CRT				
23.22 (− 83.81, 136.24)	**138.10 (33.14, 256.81)**	DIBH-IMRT			
** − 122.19 (− 252.12, − 16.80)**	− 7.54 (− 136.77, 104.75)	**− 144.92 (− 247.06, − 75.89)**	FB-IMRT		
− 34.83 (− 146.11, 90.90)	80.59 (− 33.78, 214.98)	− 57.57 (− 183.07, 76.21)	89.16 (− 34.92, 241.88)	DIBH-VMAT	
** − 164.57 (− 295.66, − 30.23)**	− 49.97 (− 180.14, 92.75)	**− 188.02 (− 332.13, − 44.95)**	− 41.42 (− 183.73, 123.92)	**− 131.12 (− 268.49, − 1.05)**	FB-VMAT
LAD mean dose					
DIBH-3D-CRT					
** − 1128.09 (− 1521.50, − 784.02)**	FB-3D-CRT				
566.27 (− 70.97, 1284.59)	**1705.76 (1051.52, 2483.06)**	DIBH-IMRT			
− 404.43 (− 1077.01, 300.80)	**737.16 (74.02, 1517.00)**	**− 970.57 (− 1429.42, − 535.45)**	FB-IMRT		
** 1648.16 (705.99, 2571.02)**	**2764.13 (1816.11, 3784.15)**	**1061.69 (305.29, 1797.44)**	**2026.80 (1293.83, 2735.46)**	DIBH-VMAT	
932.41 (− 4.68, 1870.29)	**2053.69 (1112.80, 3055.56)**	362.07 (− 436.32, 1065.41)	**1331.20 (573.79, 2039.32)**	**− 711.22 (− 1504.69, − 48.92)**	FB-VMAT
Ipsilateral lung mean dose					
DIBH-3D-CRT					
** − 140.91 (− 235.98, − 32.62)**	FB-3D-CRT				
146.28 (− 29.22, 332.94)	**287.67 (104.17, 471.32)**	DIBH-IMRT			
27.04 (− 164.38, 208.37)	168.25 (− 28.42, 348.85)	**− 119.25 (− 248.28, − 12.12)**	FB-IMRT		
259.43 (− 4.12, 535.20)	**400.40 (125.73, 672.84)**	114.51 (− 102.87, 335.73)	**233.98 (15.72, 466.67)**	DIBH-VMAT	
260.25 (− 10.02, 548.57)	**401.65 (125.52, 685.95)**	115.71 (− 109.10, 339.97)	**235.71 (22.91, 469.12)**	**− 0.42 (− 227.38, − 214.29)**	FB-VMAT
Ipsilateral lung V20					
DIBH-3D-CRT					
** − 2.43 (− 4.19, − 0.62)**	FB-3D-CRT				
1.94 (− 1.66, 5.58)	**4.39 (0.78, 8.07)**	DIBH-IMRT			
− 0.94 (− 4.64, 2.69)	1.46 (− 2.28, 5.20)	**− 2.90 (− 5.02, − 0.87)**	FB-IMRT		
** 6.40 (0.86, 12.02)**	**8.84 (3.19, 14.51)**	4.45 (0.06, 8.84)	**7.31 (2.93, 11.82)**	DIBH-VMAT	
** 5.52 (0.02, 10.99)**	**7.98 (2.37, 13.51)**	3.58 (− 0.93, 7.96)	**6.48 (1.95, 10.93)**	**− 0.85 (− 5.58, − 3.73)**	FB-VMAT

### Rank probabilities

**Figure 7:**
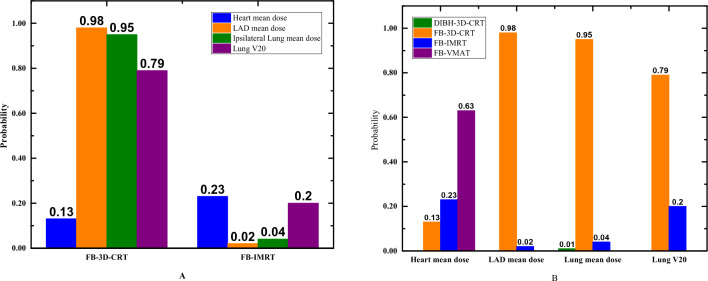
Distribution of probabilities of each agent being ranked the first place based on network (**A** represents the intercomparison of four indicators within different treatment techniques. **B** represents the comparison between different treatment techniques for the same indicator).

Figure 7 presents a ranking that indicates the probability of being the best treatment, second best, third best, and so on, among all the therapy regimens. Agents with higher values in the histogram were associated with greater probabilities for worse outcomes. Based on the network, the cumulative probability of being the most intrusive treatment in the dosimetric index were (heart mean dose, LAD mean dose, lung mean dose, lung V20): DIBH-3D-CRT (0, 0, 1%, NA), FB-3D-CRT (13%, 98%, 95%, 79%), DIBH-IMRT (0, 0, 0, 0) FB-IMRT (23%, 2%, 4%, 20%), DIBH-VMAT (0, 0, 0, 0), FB-VMAT (63%, 0, 0, 0). The numbers in brackets represent the heart mean dose, LAD mean dose, ipsilateral lung mean dose, and ipsilateral lung V20, respectively (Table 4). As the histogram in Fig. 7 illustrates, FB-3D-CRT ranked highest among all the regimens in terms of LAD mean dose, ipsilateral lung mean dose, and ipsilateral lung V20, suggesting that the FB-3D-CRT regimen is the least desirable. Moreover, FB-IMRT ranks second among all regimens in terms of LAD mean dose, ipsilateral mean dose, and ipsilateral lung V20, indicating that it is superior only to the FB-3D-CRT regimen and is inferior to even the DIBH-3D-CRT scheme. For average cardiac dose, the graph shows that FB-VMAT is the least preferred approach. Additionally, it is impossible to compare the three schemes DIBH-3D-CRT, DIBH-IMRT, and DIBH-VMAT. The detailed rank probabilities of each treatment for different outcomes are summarized in Table 4.

**Table 4:** Rank probabilities of each plan for different outcomes based on network.

Plan						
	Rank 1	Rank 2	Rank 3	Rank 4	Rank 5	Rank 6
Heart mean dose						
DIBH-3D-CRT	0.00	0.00	0.02	0.21	0.5	0.28
FB-3D-CRT	0.13	0.38	0.43	0.06	0.00	0.00
DIBH-IMRT	0.00	0.00	0.01	0.11	0.27	0.61
FB-IMRT	0.23	0.38	0.31	0.07	0.01	0.00
DIBH-VMAT	0.00	0.03	0.10	0.54	0.21	0.12
FB-VMAT	0.63	0.20	0.13	0.02	0.01	0.00
LAD mean dose						
DIBH-3D-CRT	0.00	0.11	0.84	0.04	0.01	0.00
FB-3D-CRT	0.98	0.02	0.00	0.00	0.00	0.00
DIBH-IMRT	0.00	0.00	0.03	0.82	0.15	0.00
FB-IMRT	0.02	0.87	0.11	0.00	0.00	0.00
DIBH-VMAT	0.00	0.00	0.00	0.00	0.03	0.96
FB-VMAT	0.00	0.00	0.02	0.13	0.81	0.03
Ipsilateral lung mean dose						
DIBH-3D-CRT	0.01	0.62	0.31	0.04	0.02	0.01
FB-3D-CRT	0.95	0.04	0.01	0.00	0.00	0.00
DIBH-IMRT	0.00	0.01	0.04	0.76	0.15	0.05
FB-IMRT	0.04	0.32	0.61	0.03	0.00	0.00
DIBH-VMAT	0.00	0.01	0.02	0.08	0.41	0.48
FB-VMAT	0.00	0.01	0.02	0.09	0.42	0.47
Lung V20						
DIBH-3D-CRT	0.00	0.29	0.57	0.11	0.02	0.01
FB-3D-CRT	0.79	0.19	0.01	0.00	0.00	0.00
DIBH-IMRT	0.00	0.01	0.11	0.82	0.05	0.01
FB-IMRT	0.20	0.51	0.29	0.01	0.00	0.00
DIBH-VMAT	0.00	0.00	0.01	0.02	0.33	0.65
FB-VMAT	0.00	0.00	0.01	0.04	0.60	0.34

## Discussion

### Main findings

In this study, a Bayesian network analysis of radiation therapy in cancer patients included data from twelve clinical trials, involving 714 patients with left-sided breast cancer who were assigned to six distinct treatment protocols (DIBH-3D-CRT, FB-3D-CRT, DIBH-IMRT, FB-IMRT, DIBH-VMAT, FB-VMAT). The evidence generally had NOS quality scores greater than 6. To our knowledge, this analysis was the first to use appropriate statistical methods to indirectly compare currently available regimens for the treatment of patients with left-sided breast cancer based on all available information from existing studies.

## Principal findings and comparison with other studies

Our findings elucidated the advantages and disadvantages of various radiotherapy regimens in reducing radiation doses to sensitive organs and provided clinical evidence for the promotion of DIBH technology. Two direct meta-analyses^30,31^ comparing the FB and DIBH regimens revealed that DIBH was more effective than FB in lowering the cardiac dose, left anterior descending coronary dose, and left lung dose in patients with left-sided breast cancer. These two studies provided strong support for the conclusion that DIBH is superior to FB when utilizing the same radiation modality (VMAT, IMRT, or 3D-CRT). Another recent meta-analysis^32^ that evaluated cardiac dosage and ipsilateral lung dose in patients with free-breathing in different positions (prone and supine) and deep inspiratory breath-holding demonstrated that the deep breath-holding strategy in the prone position offered the benefit of reduced organ damage. The focus of this study was on the choice of radiotherapy technology, not the radiotherapy position, hence we could not effectively support this claim.

The results of eight trials^20–23,25,27–29^ comparing DIBH-3D-CRT with FB-3D-CRT regimens showed that DIBH combined with 3D-CRT technology reduced cardiac and pulmonary dose more effectively than FB, which is consistent with the findings of this study. Besides, one trial^21^ asserted that DIBH should be prioritized in breast cancer patients who have undergone mastectomy over those who have received breast-conserving surgery. Although this aspect was not addressed in our study, we believe, from a radiological physics standpoint, that their conclusion is supported by the fact that the target area in patients after mastectomy is less physiologically curved than after breast-conserving surgery. Therefore, this is more conducive to the placement of tangential fields in the 3D-CRT plan.

Five studies^8,19,20,24,26^ from different medical institutions reported dosimetric differences between DIBH-IMRT and FB-IMRT regimens, and the results supported our Bayesian analysis that DIBH-IMRT was considerably superior to FB-IMRT in reducing the mean cardiac dose, mean LAD dose, mean pulmonary dose, and pulmonary V20. Additionally, two studies^24,29^ confirmed the finding that the DIBH-VMAT regimen is better than FB-VMAT in terms of cardiac mean dose reduction.

The previous two paragraphs only confirm that DIBH is superior to FB in terms of dosimetric metrics, given the same choice of planning modality, which is the currently prevailing perception. However, it remains to be established how 3D-CRT, IMRT, and VMAT should be chosen in clinical practice when DIBH and FB are the only options. Also, we should determine if there are any scenarios in which FB is preferable to DIBH. These issues are elaborated on below. One study^20^ that directly compared four treatment protocols: DIBH-3D-CRT, DIBH-IMRT, FB-3D-CRT, and FB-IMRT, established that DIBH-IMRT performed better than the other three protocols in terms of lowering the mean cardiac dose, LAD, and ipsilateral lung dose. Nevertheless, the results of this study did not fully advocate this conclusion, as the available data did not support the claim that DIBH-IMRT is superior to DIBH-3D-CRT in terms of cardiac and pulmonary doses. Instead, this study revealed that DIBH-3D-CRT was superior to FB-IMRT in terms of mean cardiac dose reduction, but the two studies agreed that FB-3D-CRT was the least desirable approach. This suggests that perhaps good respiratory motion management can compensate for differences in technique, and conversely, good technique can compensate for differences in respiratory motion management. However, subsequent controlled trials are still required to confirm this finding.

A direct comparison of four regimens^24^, DIBH-VMAT, DIBH-IMRT, FB-VMAT, and FB-IMRT, was also reported in the literature. It showed no difference between DIBH-VMAT and DIBH-IMRT in terms of mean cardiac dose, which was consistent with our study. Additionally, the article further stated that DIBH-VMAT was beneficial to a subset of individuals. When the mean heart dose was greater than 6.3 Gy with DIBH-IMRT, DIBH-VMAT reduced the mean heart dose^24^. However, more data are still needed to support the clinical generalizability of the 6.3 Gy threshold, which will lead to more precise and detailed studies on dosimetric comparisons in the future.

Corradini et al.^29^ performed a rigorous dosimetric comparison of four regimens: DIBH-3D-CRT, DIBH-VMAT, FB-3D-CRT, and FB-VMAT, and conducted a corresponding risk assessment for the development of secondary lung cancer and ischemic heart disease. According to their findings, DIBH-3D-CRT correlated with the lowest incidence of major coronary events and secondary lung cancer. However, our findings differed greatly from those of Corradini et al. In terms of average cardiac dose, our results suggested that DIBH-3D-CRT was superior to FB-VMAT and FB-3D-CRT, but not DIBH-VMAT. Similarly, in terms of average pulmonary dose, our results did not indicate that DIBH-3D-CRT was the optimal choice among the four plans. It should be noted that Corradini et al.^29^ used dual energies of 6 MV and 15 MV and the collapsed cone algorithm for the 3D-CRT scheme in their study, while a single energy of 6 MV and the Monte Carlo algorithm were used in the VMAT scheme. In our work, the different choices of energy and algorithm may have led to differences in dose results, thereby contributing to the discrepancies in the conclusions between their study and ours. Furthermore, a similar study was conducted by Osman et al.^33^ and their results showed that DIBH-VMAT was superior to DIBH-3D-CRT, which contradicts the results of Corradini et al.^29^. Based on this, further controlled studies are still required to produce more definitive results.

## Limitations of this study

The main limitation of this study was the inclusion of a single study protocol, all with a 50 GY/25 score, which led to the inclusion of only a small number of studies. Studies concerning VMAT and IMRT were far fewer than those of 3D-CRT. However, this was a necessary trade-off made to ensure that the study baselines were as consistent as possible. Additionally, there was an unavoidable degree of heterogeneity between the studies, such as patient position design, options for treatment planning systems, selection of planning algorithms, and the breath-holding methods employed by patients during DIBH. Since all the included literature was analysed in comparison to the crisis organ receptivity with sufficient dose in the target area, the conformity index (CI) and homogeneity index (HI) of the target area were not critically evaluated again in this paper. Furthermore, the evaluation of pulmonary dosimetric parameters, such as V20 and mean dosage. Because the lung parenchymal density (total number of alveoli divided by total lung volume) in DIBH may be smaller, these metrics may not be biologically similar in DIBH vs FB designs, and this is not addressed in depth in this article.

## Conclusions

In circumstances when the treatment plan is predetermined, we discovered that the DIBH approach should be utilized as the op-timal method. A comprehensive evaluation of the cardiopulmonary dose of the six regimens revealed that FB-3D-CRT is probably the least attractive option, while the priority of the other five regimens should be determined based on the patient's actual cardiopulmo-nary function and which organ the clinician believes is most at risk, according to the dosimetric index.

## Data Availability

The original contributions presented in the study are included in the article/supplementary material. Further inquiries can be directed to the corresponding author.
